# Supplementary material for: Structural basis for pegRNA-guided reverse transcription by a prime editor
Source: Nature. 2024 May 29;631(8019):224–31. doi: 10.1038/s41586-024-07497-8 (PMC11222144; doi:10.1038/s41586-024-07497-8)
Supplement: Supplementary file 1 — This file contains Supplementary Figures 1–3 and Supplementary Tables 1–3 [file 41586_2024_7497_MOESM1_ESM.pdf]

nature portfolio

https://doi.org/10.1038/s41586-024-07497-8

Supplementary information

Structural basis for pegRNA-guided reverse transcription by a prime editor

In the format provided by the authors and unedited

Supplementary Information for

Structural basis for pegRNA-guided reverse transcription by prime editor

Yutaro Shuto[1,7], Ryoya Nakagawa[1,7*], Shiyou Zhu[2,3,4,5,6], Mizuki Hoki[1], Satoshi N. Omura[1], Hisato Hirano[1], Yuzuru Itoh[1], Feng Zhang[2,3,4,5,6], and Osamu Nureki[1*]

*Correspondence: ryoya.nakagawa@bs.s.u-tokyo.ac.jp, nureki@bs.s.u-tokyo.ac.jp

This PDF file includes:

Supplementary Figs. 1, 2, 3
Supplementary Tables 1, 2, 3

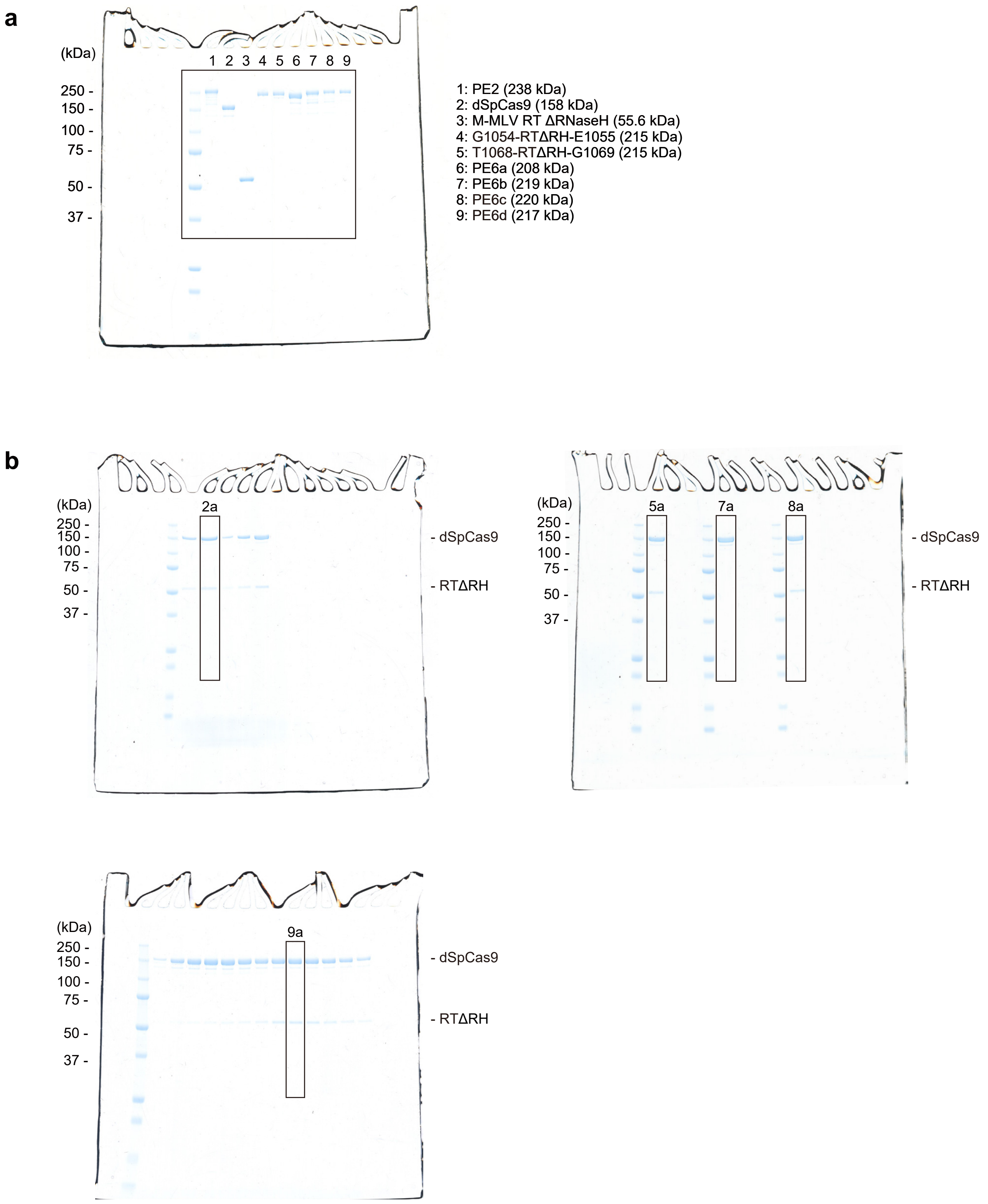

Supplementary Fig. 1. Uncropped SDS-PAGE gel images in this study.

a, SDS-PAGE gel of purified proteins (used for Extended Data Fig. 1a).

b, SDS-PAGE gels of prime editor complex reconstitutions. After size-exclusion chromatography, the peak fraction was analysed (used for Extended Data Fig. 2a, 5a, 7a, 8a, and 9a).

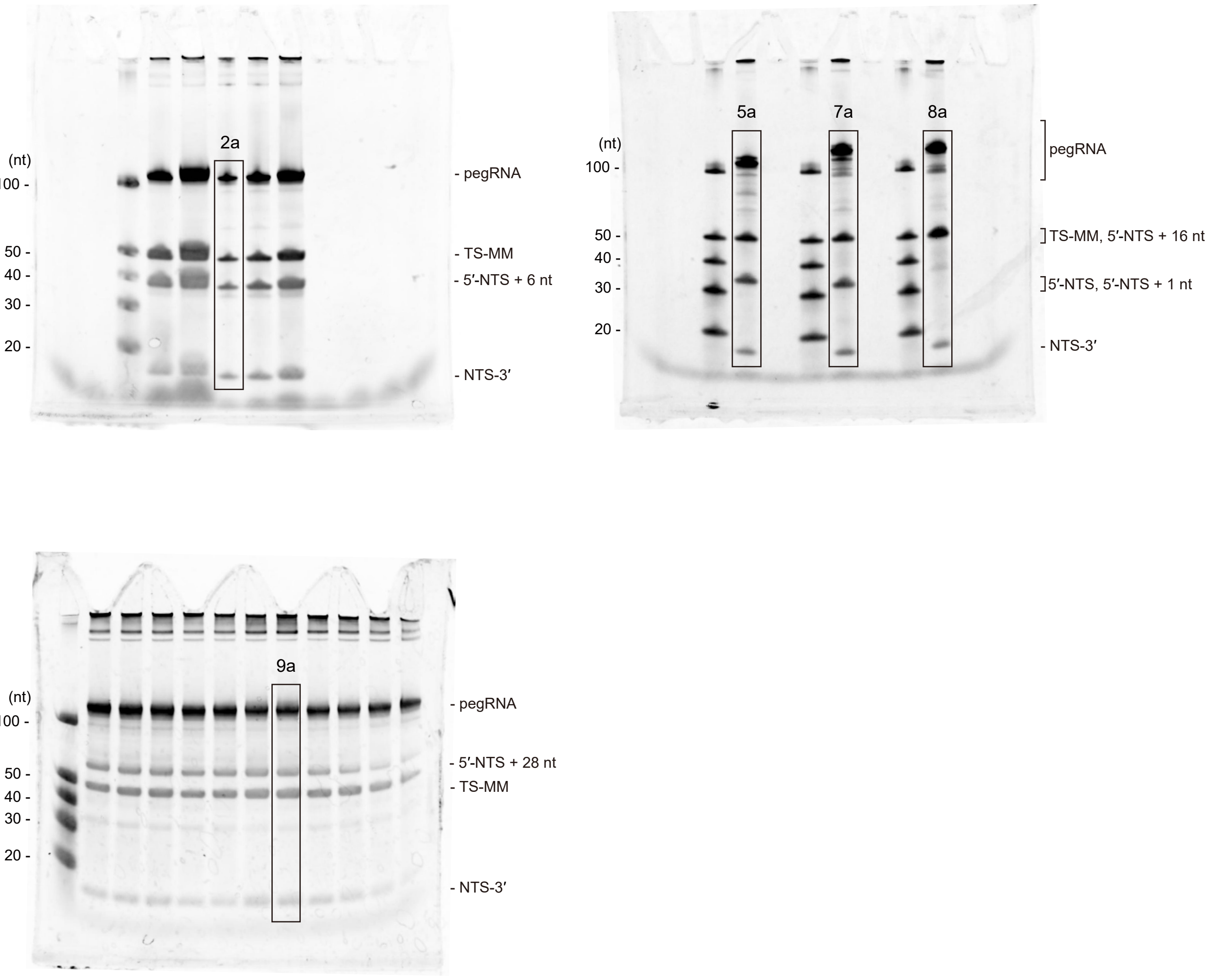

Supplementary Fig. 2. Uncropped urea-PAGE gel images of prime editor complex reconstitutions. After size-exclusion chromatography, the peak fraction was analysed. The gels were visualized by SYBR Gold staining (used for Extended Data Fig. 2a, 5a, 7a, 8a, and 9a).

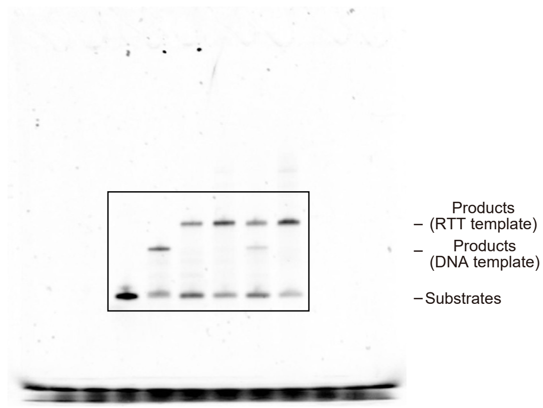

Fig. 1c

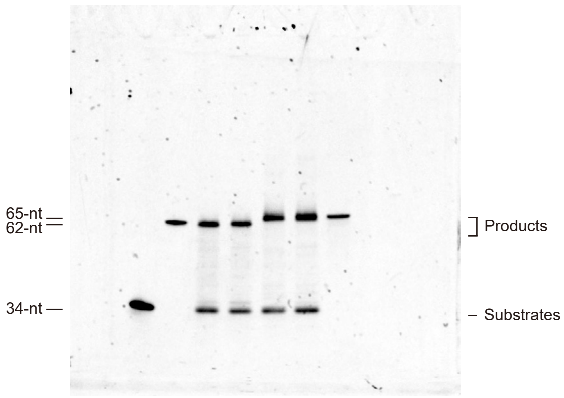

Fig. 3c

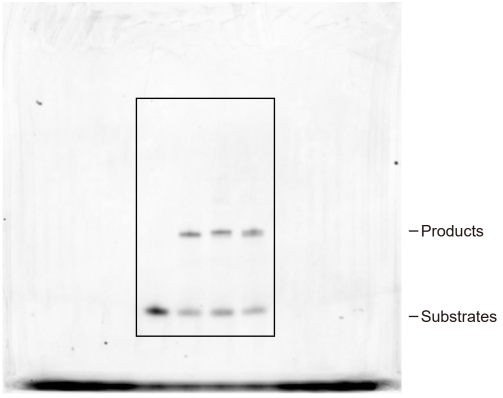

Fig. 4h

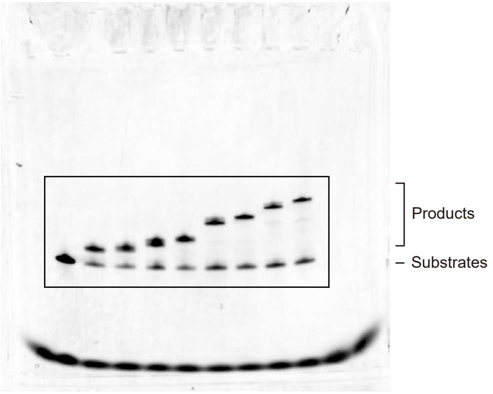

Extended Data Fig. 4a

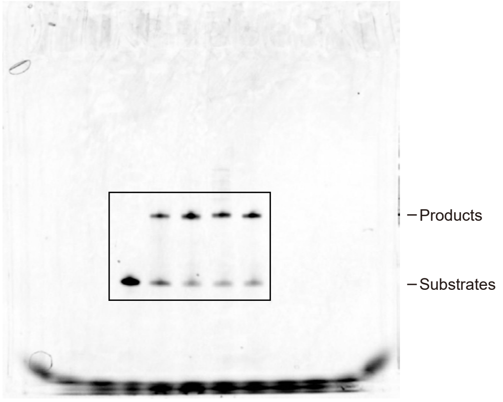

Extended Data Fig. 4d

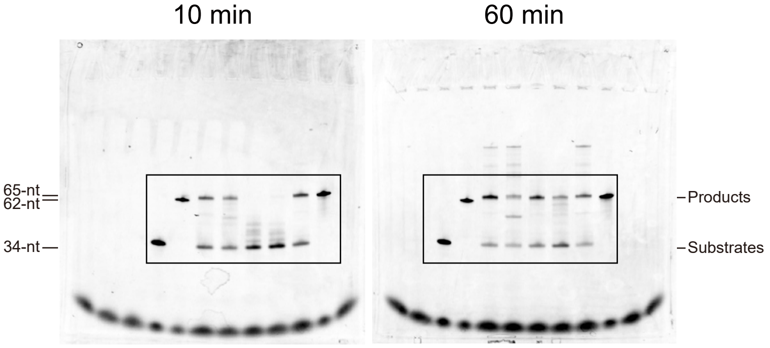

Extended Data Fig. 10f

Supplementary Fig. 3. Uncropped urea-PAGE gel images of *in vitro* prime editing assay. After the reactions with Cy5-labeled substrates were stopped by the addition of quench buffer, the reaction products were separated on urea-PAGE gels. The gels were visualized by Cy5 fluorescence (used for Fig. 1c, 3c, and 4h and Extended Data Fig. 4a, 4d, and 10f).

Supplementary Table 1. Nucleic-acid sequences used in prime editing assay.

In vitro prime editing assay

Target DNA

Name	Sequence
TS	CTAGTACTCTGCCATCACGTGCTCAGTCTGGGCCGATTGCTCTAAGTGATC
TS-MM	CTAGTACTCTGCCATCAGAGCAAGCACTACGGCCGATTGCTCTAAGTGATC
5′-Cy5-NTS	GATCACTTAGAGCAATCGGCCCAGACTGAGCACG
NTS-3′	TGATGGCAGAGTACTAG

pegRNA

Name	Sequence
pegRNA	GGCCCAGACUGAGCACGUGAguuuuagagcuagaaauagcaaguuaaaauaaggcuaguccguuaucaacuugaaaaaguggcaccgagucggugcUGGAGGAAGCAGGGCAACCAAACCACAGCGUGCUCAGUCUG
pegRNA-MM	GGCC**GUAGUGCUUGCUC**UGAguuuuagagcuagaaauagcaaguuaaaauaaggcuaguccguuaucaacuugaaaaaguggcaccgagucggugcUGGAGGAAGCAGGGCAACCAAACCACAGCGUGCUCAGUCUG
pegRNA-6nt	GGCCCAGACUGAGCACGUGAguuuuagagcuagaaauagcaaguuaaaauaaggcuaguccguuaucaacuugaaaaaguggcaccgagucggugcUCACAGCGUGCUCAGUCUG
pegRNA-10nt	GGCCCAGACUGAGCACGUGAguuuuagagcuagaaauagcaaguuaaaauaaggcuaguccguuaucaacuugaaaaaguggcaccgagucggugcUGGAGGAAGCCGUGCUCAGUCUG
pegRNA-20nt	GGCCCAGACUGAGCACGUGAguuuuagagcuagaaauagcaaguuaaaauaaggcuaguccguuaucaacuugaaaaaguggcaccgagucggugcUGGAGGAAGCAGGGCAACCACGUGCUCAGUCUG
pegRNA-ACG	GGCCCAGACUGAGCACGUGAguuuuagagcuagaaauagcaaguuaaaauaaggcuaguccguuaucaacuugaaaaagug**CGU**ccgagucgg**ACG**UGGAGGAAGCAGGGCAACCAAACCACAGCGUGCUCAGUCUG
pegRNA-AAA	GGCCCAGACUGAGCACGUGAguuuuagagcuagaaauagcaaguuaaaauaaggcuaguccguuaucaacuugaaaaagug**UUU**ccgagucgg**AAA**UGGAGGAAGCAGGGCAACCAAACCACAGCGUGCUCAGUCUG
pegRNA-GGG	GGCCCAGACUGAGCACGUGAguuuuagagcuagaaauagcaaguuaaaauaaggcuaguccguuaucaacuugaaaaagug**CCC**ccgagucgg**GGG**UGGAGGAAGCAGGGCAACCAAACCACAGCGUGCUCAGUCUG

The guide sequence in the pegRNAs and the target sequence in the TS are underlined, and the scaffold region in the pegRNA is in lowercase. Moreover, in the lower three modified pegRNAs, the different sequences from the pegRNA are highlighted in bold or uppercase.The RTT sequences are colored yellow. The PBS sequences and their complementary sequences in the NTS are colored pink. The PAM sequences (TGG) in the NTS are colored purple.

In vivo prime editing assay

pegRNA or nicking sgRNA

Edit		Sequence
HEK3 +1 T to A	pegRNA-WT	GGCCCAGACUGAGCACGUGAguuuuagagcuagaaauagcaaguuaaaauaaggcuaguccguuaucaacuugaaaaaguggcaccgagucggugcUCCUCUGCCAUCUCGUGCUCAGUCUG
	pegRNA-mutant	GGCCCAGACUGAGCACGUGAguuuuagagcuagaaauagcaaguuaaaauaaggcuaguccguuaucaacuugaaaaagug**AAG**ccgagucgg**CUU**UCCUCUGCCAUCUCGUGCUCAGUCUG
EMX1 +1 G to C	pegRNA-WT	GAGUCCGAGCAGAAGAAGAAguuuuagagcuagaaauagcaaguuaaaauaaggcuaguccguuaucaacuugaaaaaguggcaccgagucggugcGUGAUGGGAGCCCUUGUUCUUCUGCUCGG
	pegRNA-mutant	GAGUCCGAGCAGAAGAAGAAguuuuagagcuagaaauagcaaguuaaaauaaggcuaguccguuaucaacuugaaaaagug**AUC**ccgagucgg**GAU**GUGAUGGGAGCCCUUGUUCUUCUGCUCGG
HEK3 +1 A insert	pegRNA-WT	GGCCCAGACUGAGCACGUGAguuuuagagcuagaaauagcaaguuaaaauaaggcuaguccguuaucaacuugaaaaaguggcaccgagucggugcUCUGCCAUCACGUGCUCAGUCUG
	pegRNA-mutant	GGCCCAGACUGAGCACGUGAguuuuagagcuagaaauagcaaguuaaaauaaggcuaguccguuaucaacuugaaaaagug**GGA**ccgagucgg**UCC**UCUGCCAUCACGUGCUCAGUCUG
FANCF +5 G to C	pegRNA-WT	GGAAUCCCUUCUGCAGCACCguuuuagagcuagaaauagcaaguuaaaauaaggcuaguccguuaucaacuugaaaaaguggcaccgagucggugcGGAAAAGCGAUCAAGGUGCUGCAGAAGGGA
	pegRNA-mutant	GGAAUCCCUUCUGCAGCACCguuuuagagcuagaaauagcaaguuaaaauaaggcuaguccguuaucaacuugaaaaagug**GAG**ccgagucgg**CUC**GGAAAAGCGAUCAAGGUGCUGCAGAAGGGA
RNF2 +1 C to A	pegRNA-WT	GUCAUCUUAGUCAUUACCUGguuuuagagcuagaaauagcaaguuaaaauaaggcuaguccguuaucaacuugaaaaaguggcaccgagucggugcAACGAACACCUCAUGUAAUGACUAAGAUG
	pegRNA-mutant	GUCAUCUUAGUCAUUACCUGguuuuagagcuagaaauagcaaguuaaaauaaggcuaguccguuaucaacuugaaaaagug**GUA**ccgagucgg**UAC**AACGAACACCUCAUGUAAUGACUAAGAUG
FANCF +5-7 GGA del	pegRNA-WT	GGAAUCCCUUCUGCAGCACCguuuuagagcuagaaauagcaaguuaaaauaaggcuaguccguuaucaacuugaaaaaguggcaccgagucggugcGGAAAAGCGAAGGUGCUGCAGAAGGGAU
	pegRNA-mutant	GGAAUCCCUUCUGCAGCACCguuuuagagcuagaaauagcaaguuaaaauaaggcuaguccguuaucaacuugaaaaagug**GAG**ccgagucgg**CUC**GGAAAAGCGAAGGUGCUGCAGAAGGGAU
	sgRNA	GCCCUACUUCCGCUUUCACCUguuuuagagcuagaaauagcaaguuaaaauaaggcuaguccguuaucaacuugaaaaaguggcaccgagucggugc
FANCF +8 T to C	pegRNA-WT	GGAAUCCCUUCUGCAGCACCguuuuagagcuagaaauagcaaguuaaaauaaggcuaguccguuaucaacuugaaaaaguggcaccgagucggugcGGAAAAGCGGUCCAGGUGCUGCAGAAGGGAU
	pegRNA-mutant	GGAAUCCCUUCUGCAGCACCguuuuagagcuagaaauagcaaguuaaaauaaggcuaguccguuaucaacuugaaaaagug**GAG**ccgagucgg**CUC**GGAAAAGCGGUCCAGGUGCUGCAGAAGGGAU
	sgRNA	GCCCUACUUCCGCUUUCACCUguuuuagagcuagaaauagcaaguuaaaauaaggcuaguccguuaucaacuugaaaaaguggcaccgagucggugc
RNF2 +1 T ins	pegRNA-WT	GUCAUCUUAGUCAUUACCUGguuuuagagcuagaaauagcaaguuaaaauaaggcuaguccguuaucaacuugaaaaaguggcaccgagucggugcAACGAACACCUCAGAGUAAUGACUAAGAUG
	pegRNA-mutant	GUCAUCUUAGUCAUUACCUGguuuuagagcuagaaauagcaaguuaaaauaaggcuaguccguuaucaacuugaaaaagug**GUA**ccgagucgg**UAC**AACGAACACCUCAGAGUAAUGACUAAGAUG
	sgRNA	UCAACCAUUAAGCAAAACAUguuuuagagcuagaaauagcaaguuaaaauaaggcuaguccguuaucaacuugaaaaaguggcaccgagucggugc
RNF2 +4 A del	pegRNA-WT	GGAAUCCCUUCUGCAGCACCguuuuagagcuagaaauagcaaguuaaaauaaggcuaguccguuaucaacuugaaaaaguggcaccgagucggugcAACGAACACCCAGGUAAUGACUAAGAUG
	pegRNA-mutant	GUCAUCUUAGUCAUUACCUGguuuuagagcuagaaauagcaaguuaaaauaaggcuaguccguuaucaacuugaaaaagug**GUA**ccgagucgg**UAC**AACGAACACCCAGGUAAUGACUAAGAUG
	sgRNA	UCAACCAUUAAGCAAAACAUguuuuagagcuagaaauagcaaguuaaaauaaggcuaguccguuaucaacuugaaaaaguggcaccgagucggugc

The guide sequence in the pegRNAs is underlined, and the scaffold region in the pegRNA is in lowercase. Moreover, the different sequences in the mutants from their WT are highlighted in bold and uppercase.The RTT sequences are colored yellow. The PBS sequences are colored pink.

Supplementary Table 2. Nucleic-acid sequences used in Cryo-EM analysis.

State	Name	Sequence
Termination	pegRNA-termination-MM	GGCCGUAGUGCUUGCUCUGAguuuuagagcuagaaauagcaaguuaaaauaaggcuaguccguuaucaacuugaaaaaguggcaccgagucggugc**U**CACAGCGUGCUCAGUCUG
	5′-NTS+3nt	CTAGTACTCTGCCATCAGAGCAAGCACTACGGCCGATTGCTCTAAGTGATC
	TS-MM	GATCACTTAGAGCAATCGGCCCAGACTGAGCACGCTG
	NTS-3′	TGATGGCAGAGTACTAG
Initiation	pegRNA-initiation-MM	GGCCGUAGUGCUUGCUCUGAguuuuagagcuagaaauagcaaguuaaaauaaggcuaguccguuaucaacuugaaaaaguggcaccgagucggugcGCACA**U**CGUGCUCAGUCUG
	TS-MM	CTAGTACTCTGCCATCAGAGCAAGCACTACGGCCGATTGCTCTAAGTGATC
	5′-NTS	GATCACTTAGAGCAATCGGCCCAGACTGAGCACG
	NTS-3′	TGATGGCAGAGTACTAG
Pre-initiation	pegRNA-pre-initiation-MM	GGCCGUAGUGCUUGCUCUGAguuuuagagcuagaaauagcaaguuaaaauaaggcuaguccguuaucaacuugaaaaaguggcaccgagucggugcAGGAGGAAGCAGUGCAACCAAACCACAGCGUGCUCAGUCUG
	TS-MM	CTAGTACTCTGCCATCAGAGCAAGCACTACGGCCGATTGCTCTAAGTGATC
	5′-NTS	GATCACTTAGAGCAATCGGCCCAGACTGAGCACG
	NTS-3′	TGATGGCAGAGTACTAG
Elongation (16 nt)	pegRNA-elongation-16nt-MM	GGCCGUAGUGCUUGCUCUGAguuuuagagcuagaaauagcaaguuaaaauaaggcuaguccguuaucaacuugaaaaaguggcaccgagucggugcAGGAGGAAGCAG**U**GCAACCAAACCACAGCGUGCUCAGUCUG
	TS-MM	CTAGTACTCTGCCATCAGAGCAAGCACTACGGCCGATTGCTCTAAGTGATC
	5′-NTS+3nt	GATCACTTAGAGCAATCGGCCCAGACTGAGCACGCTG
	NTS-3′	TGATGGCAGAGTACTAG
Elongation (28 nt)	pegRNA-elongation-28nt-MM	GGCCGUAGUGCUUGCUCUGAguuuuagagcuagaaauagcaaguuaaaauaaggcuaguccguuaucaacuugaaaaaguggcaccgagucggugc**U**GGAGGAAGCAGGGCAACCAAACCACAGCGUGCUCAGUCUG
	TS-MM	CTAGTACTCTGCCATCAGAGCAAGCACTACGGCCGATTGCTCTAAGTGATC
	5′-NTS+3nt	GATCACTTAGAGCAATCGGCCCAGACTGAGCACGCTG
	NTS-3′	TGATGGCAGAGTACTAG

The guide sequence in the pegRNAs and the target sequence in the TS are underlined, and the scaffold region in the pegRNA is in lowercase. The RTT sequences are colored in yellow, with uracil in the RTT highlighted in bold except for the pegRNA-pre-initiation-MM. The PBS sequences and their complementary sequences in the NTS are colored pink. The reverse transcription sequences pre-added to the NTS are colored in blue. The PAM sequences (TGG) in the NTS are colored purple.

Supplementary Table 3. Oligonucleotides and constructs used in this study.

Mutants

Mutation		Forward primer	Reverse primer
D10A		GCCATCGGCACCAACTCTGTGGGC	CAGGCCGATGCTGTACTTCTTGTCACCTCCA
G1054-RTΔRH-E1055	insertion	ACCCTGGCCAACGGCTCCGGGACAGGGGGTACCCTAAATATAGAAGATGAGTATCGGCTACATG	CCGCTTCCGGATCTCTCCCGACCCCGTTCCAAGGCAGTTGTGTTGCAGCCCTTC
	vector	GAGATCCGGAAGCGGCCTCTGATCGAGAC	GCCGTTGGCCAGGGTAATCTCGGTCTTG
T1068-RTΔRH-G1069	insertion	ACAAACGGCGAAACCTCCGGGACAGGGGGTACCCTAAATATAGAAGATGAGTATCGGCTACATG	CCACACGATCTCCCCTCCCGACCCCGTTCCAAGGCAGTTGTGTTGCAGCCCTTC
	vector	GGTTTCGCCGTTTGTCTCGATCAGAGGCCGCTTC	GCCGTTGGCCAGGGTAATCTCGGTCTTG

Constructs

Plasmid	
His-SUMO-PE2	https://benchling.com/s/seq-NSua7gucIIlgrqjbUgTH?m=slm-f2zy3CFR9yplWc0x6aoS
His-SUMO-SpCas9 D10A/H840A	https://benchling.com/s/seq-PKZ29WQG5SLvXMUFvTH4?m=slm-j9XhGzQ0hVi92MwJrd86
His-SUMO-RTΔRH	https://benchling.com/s/seq-5spL3SO3h9IYxs3bsWFa?m=slm-YahBQRVNgYQS8mvqXRtr
His-SUMO-G1054-RTΔRH-E1055	https://benchling.com/s/seq-OyRwnDSdsOuam6NtNa3G?m=slm-w3oQgNCixA32chdwWUgy
His-SUMO-T1068-RTΔRH-G1069	https://benchling.com/s/seq-To7DIVFp4fkK7Jaa4z6P?m=slm-wW8hClEjSSDEHseFwMiI
His-SUMO-PE6a	https://benchling.com/s/seq-tjRzTiRgBo3mwWaxiPrw?m=slm-q6276dfnucvtCsm1LVrE
His-SUMO-PE6b	https://benchling.com/s/seq-tWyqrcDfQLRmgT8xx3tA?m=slm-1dsj1kE73NEwo2rv6S7G
His-SUMO-PE6c	https://benchling.com/s/seq-7cXy6MwkHCLeGRG8U495?m=slm-c43MX30JwkzXID5TCUtQ
His-SUMO-PE6d	https://benchling.com/s/seq-0pmF3oRQxiEX6avdostb?m=slm-FFHeOcVK2GMAUUAbhi4A

In vitro transcription templates

Forward template	Sequence
T7promoter-Fw	GGATCCTAATACGACTCACTATA

Reverse template	Sequence
pegRNA-Rv	CAGACTGAGCACGCTGTGGTTTGGTTGCCCTGCTTCCTCCAgcaccgactcggtgccactttttcaagttgataacggactagccttattttaacttgctatttctagctctaaaacTCACGTGCTCAGTCTGGGCCTATAGTGAGTCGTATTAGGATCC
pegRNA-MM-Rv	CAGACTGAGCACGCTGTGGTTTGGTTGCCCTGCTTCCTCCAgcaccgactcggtgccactttttcaagttgataacggactagccttattttaacttgctatttctagctctaaaacTCAGAGCAAGCACTACGGCCTATAGTGAGTCGTATTAGGATCC
pegRNA-6nt-Rv	CAGACTGAGCACGCTGTGAgcaccgactcggtgccactttttcaagttgataacggactagccttattttaacttgctatttctagctctaaaacTCACGTGCTCAGTCTGGGCCTATAGTGAGTCGTATTAGGATCC
pegRNA-10nt-Rv	CAGACTGAGCACGGCTTCCTCCAgcaccgactcggtgccactttttcaagttgataacggactagccttattttaacttgctatttctagctctaaaacTCACGTGCTCAGTCTGGGCCTATAGTGAGTCGTATTAGGATCC
pegRNA-20nt-Rv	CAGACTGAGCACGTGGTTGCCCTGCTTCCTCCAgcaccgactcggtgccactttttcaagttgataacggactagccttattttaacttgctatttctagctctaaaacTCACGTGCTCAGTCTGGGCCTATAGTGAGTCGTATTAGGATCC
pegRNA-ACG-Rv	CAGACTGAGCACGCAGAGGAAAGGAAGCCCTGCTTCCTCCAcgtccgactcggacgcactttttcaagttgataacggactagccttattttaacttgctatttctagctctaaaacTCACGTGCTCAGTCTGGGCCTATAGTGAGTCGTATTAGGATCC
pegRNA-AAA-Rv	CAGACTGAGCACGCAGAGGAAAGGAAGCCCTGCTTCCTCCAtttccgactcggaaacactttttcaagttgataacggactagccttattttaacttgctatttctagctctaaaacTCACGTGCTCAGTCTGGGCCTATAGTGAGTCGTATTAGGATCC
pegRNA-GGG-Rv	CAGACTGAGCACGCAGAGGAAAGGAAGCCCTGCTTCCTCCAcccccgactcgggggcactttttcaagttgataacggactagccttattttaacttgctatttctagctctaaaacTCACGTGCTCAGTCTGGGCCTATAGTGAGTCGTATTAGGATCC
pegRNA-termination-MM-Rv	CAGACTGAGCACGCTGTGAgcaccgactcggtgccactttttcaagttgataacggactagccttattttaacttgctatttctagctctaaaacTCAGAGCAAGCACTACGGCCTATAGTGAGTCGTATTAGGATCC
pegRNA-initiation-MM-Rv	CAGACTGAGCACGATGTGCgcaccgactcggtgccactttttcaagttgataacggactagccttattttaacttgctatttctagctctaaaacTCAGAGCAAGCACTACGGCCTATAGTGAGTCGTATTAGGATCC
pegRNA-pre-initiation-MM-Rv	CAGACTGAGCACGCTGTGGTTTGGTTGCACTGCTTCCTCCTgcaccgactcggtgccactttttcaagttgataacggactagccttattttaacttgctatttctagctctaaaacTCAGAGCAAGCACTACGGCCTATAGTGAGTCGTATTAGGATCC
pegRNA-elongation-16nt-MM-Rv	CAGACTGAGCACGCTGTGGTTTGGTTGCACTGCTTCCTCCTgcaccgactcggtgccactttttcaagttgataacggactagccttattttaacttgctatttctagctctaaaacTCAGAGCAAGCACTACGGCCTATAGTGAGTCGTATTAGGATCC
pegRNA-elongation-28nt-MM-Rv	CAGACTGAGCACGCTGTGGTTTGGTTGCCCTGCTTCCTCCAgcaccgactcggtgccactttttcaagttgataacggactagccttattttaacttgctatttctagctctaaaacTCAGAGCAAGCACTACGGCCTATAGTGAGTCGTATTAGGATCC

The scaffold region in the pegRNA templates is in lowercase. The sequences complementary to T7promoter-Fw in the reverse templates are underlined.
